# Relationship between modifiable lifestyle factors and chronic kidney disease: a bibliometric analysis of top-cited publications from 2011 to 2020

**DOI:** 10.1186/s12882-022-02745-3

**Published:** 2022-03-25

**Authors:** Ting Yin, Yilong Chen, Lei Tang, Huaihong Yuan, Xiaoxi Zeng, Ping Fu

**Affiliations:** 1 Division of Nephrology, Kidney Research Institute, West China Hospital of Sichuan University, 37 Guo Xue Xiang, Chengdu, China; 2 West China Biomedical Big Data Center, West China Hospital, Sichuan University, 37 Guo Xue Xiang, Chengdu, China; 3 West China School of Nursing, Sichuan University, 37 Guo Xue Xiang, Chengdu, China

**Keywords:** Chronic kidney disease, Modifiable factors, Lifestyle, Obesity, Physical activity, Diet, Bibliometric analysis

## Abstract

**Background:**

Chronic kidney disease (CKD) affects 8 to 16% of the world’s population and is one of the top ten important drivers of increasing disease burden. Apart from genetic predisposition, lifestyle factors greatly contribute to the incidence and progression of CKD. The current bibliometric analysis aims to characterize the current focus and emerging trends of the research about the impact of modifiable lifestyle factors on CKD.

**Methods:**

We searched articles addressing the impact of modifiable lifestyle factors on the incidence and/or progression of CKD, published between 2011 and 2020, from the Science Citation Index Expanded (SCIE) database. An adjusted citation index, which considered both the original citation count and publication year, was derived for the selection of most-cited publications. Publishing trends, co-authorship network, keywords, and research hotspots were analyzed and visualized.

**Results:**

Among the top 100 most influential articles, 32 were narrative reviews, 16 systematic reviews and/or meta-analysis, 44 clinical research, and 8 basic research. The United States occupied a dominant position in the perspective of article numbers and international partnerships, followed by European countries. The modifiable factors that drew the most and constant attention over the decade were diet or nutrition management reported in 63 papers, followed by obesity or body mass index (*n* = 27), and physical activity or exercises (*n* = 8). Alcohol consumption, fish oil, chain fatty-acids, and water-soluble vitamins were emerging hotspots identified in the recent most cited publications.

**Conclusions:**

Based on the bibliometric analysis of the most influential articles, our study provides a comprehensive description of publishing trends and research focus over a decade in the field of lifestyle factors’ impact on CKD. Diet, obesity, and physical activity were factors receiving the most attention in this topic.

**Supplementary Information:**

The online version contains supplementary material available at 10.1186/s12882-022-02745-3.

## Background

Chronic kidney disease (CKD), defined as abnormalities of kidney structure or function presenting for >3 months with health implications [1], affects 8–16% of the world’s population [2]. CKD is closely associated with an increased risk of adverse events, including end-stage renal disease (ESRD), cardiovascular events, hospitalizations, and mortality [3–7]. The global all-age mortality rate from CKD increased by 41.5% from 1990 to 2017 [8]. It is currently ranking the 16th leading cause of years of life lost [2] and is projected to rise in the ranking, to the 5th, by 2040 [9].

CKD imposes great burdens in both developed and developing countries. For example, Medicare expenditures for ESRD in the United States (the US) increased by about 20.3% from 2009 to 2018 and accounted for 7.2% of overall Medicare fee-for-service spending in 2018 [10]. The burden of CKD is even more pronounced in low- and lower-middle-income countries [11]. In several regions, particularly Oceania, sub-Saharan Africa, and Latin America, the burden of CKD is much higher than expected for the level of development [8]. Thus, it is considered to be one of the top ten important drivers of increasing burden according to the latest analysis for the Global Burden of Disease Study [12].

CKD is affected by both genetic and environmental factors [13]. While modification of the genetic predisposition for CKD is quite challenging, evidence shows that the incidence and rapid progression of CKD can be protected via modifiable lifestyle factors, ie. diet, physical activity, alcohol consumption, tobacco smoking, sleep, and obesity [14–18]. Recently, growing literature has addressed the underlying mechanism and the health impact of modifiable factors on CKD. However, to our knowledge, there have been no bibliometric analyses on this topic.

Bibliometric analysis is a valuable tool for navigation in a particular research area [19, 20]. It has been used to provide qualitative and quantitative analysis of publications, enabling researchers to identify core articles, study hotspots, and publishing patterns within a given subject area [21]. Thus, bibliometric analysis is an integral part of the evaluation methodology for assessment of the research evolution and current development stage of the discipline [19, 22]. In this study, based on bibliometric analysis of the top-cited articles, we aim to elucidate the current focus, growing trends, and future direction of the research about the impact of modifiable factors on CKD.

## Methods

### Search strategy

We aimed to analyze the most influential publications within the last ten years. Inclusion criteria were (1) original articles or reviews which addressed the impact of modifiable lifestyles, ie. diet (including foods, nutrients, and dietary patterns), physical activity, alcohol consumption, smoking, sleep, and obesity, on the incidence and/or progression of CKD; (2) published between January 2011 to December 2020; (3) with restriction of language of English. The Science Citation Index Expanded database (SCIE) of Web of Science, which includes multidisciplinary bibliographic information and is now regarded as one of the collections of the highest impact, most influential international and regional journals [23, 24], was searched for the inclusion of publications. A comprehensive search strategy was performed to identify the intersect of search terms describing CKD and modifiable lifestyles, being limited to the field of “Topic”. Other document types, such as meeting abstracts, letters, and corrections, were excluded. The detailed search terms were listed in Table S1.

### Study selection and data collection

Considering papers that were published earlier had innately higher opportunities to be cited than those published later, to ensure recently published influential papers also be included for analysis, we derived an adjusted citation index, which was defined as the mean number of citations per year, calculated by the equation: adjusted citation index = total cited count / (2021- publication year), to evaluate the publications for inclusion.

**Fig. 1:**
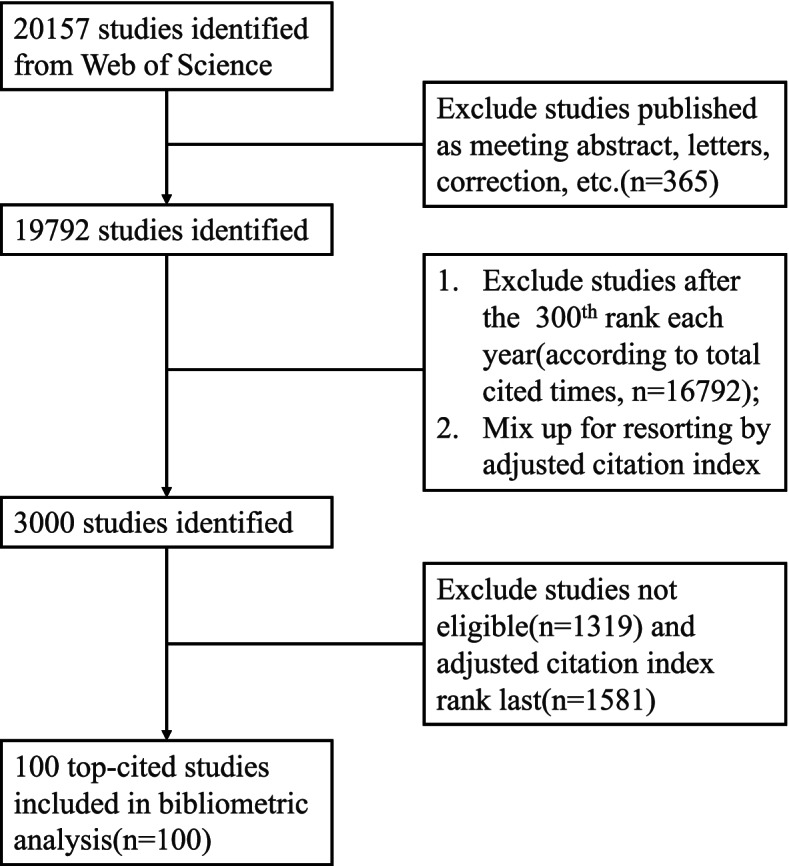
Flow diagram of the article selection process used in the study

Among the original 20,157 records obtained via the above-mentioned search strategy, to facilitate the following study selection process, we firstly excluded studies after the 300th rank each year in descending order of total citation count. Next, for the 3000 remaining studies for further evaluation, the abstracts, as well as full texts when necessary, were thoroughly reviewed by investigators (YT and HY) independently to exclude ineligible studies. Any disagreement was resolved through discussion with another viewer (XZ). Finally, the top 100-cited articles according to the adjusted citation index were included in our bibliometric analysis. The information of titles, authors, institutions, abstracts, countries, publication years, journals, total citation number of the article, document types, author keywords and keywords plus, and research areas of these publications, were downloaded on 6 August 2021. The selection process of the articles was shown in Fig. 1.

### Bibliometric analysis

We read the abstract or full text of each article and classified the research into clinical research, basic research, review, and meta-analysis. We used ‘bibliometrix’ package in R software (version 3.6.3) to analyze the bibliographic information [25]. The country of origin of the articles was defined according to the corresponding author. H-index was used to evaluate scholars’ scientific output based on their published articles and citations. The value of H-index is equal to the number of papers (N) of a researcher that has been cited by others at least N times [26]. The annual percentage growth rate was calculated by ‘biblioAnalysis’ function in R software to describe the annual change in the scientific production.

VOSviewer software (version 1.6.16) [27] was used to visualize the co-authorship network and analyze keywords. The link in the co-authorship network represented authors’ collaborations and bigger nodes indicated more publications of the authors [28].

For keyword analysis, we extracted both the original keywords provided by the authors and the keywords plus, which were words or phrases that frequently appeared in the titles of an article’s references but did not appear in the title of the article itself. For articles that did not provide author keywords, keywords plus were used instead, since keywords plus were considered to be as effective as author keywords when investigating the knowledge structure of scientific fields [29]. We merged some synonym keywords and unified keywords with the same meaning (Table S2) [30]. For example, “chronic kidney disease”, “chronic renal insufficiency”, and “CKD” were merged into “chronic kidney disease”. We derived a word cloud to visualize the word significance, the dimensions of each word representing the frequency of occurrences in publications [31]. The keyword co-occurrence network, in which an edge between two nodes representing the co-occurrence of two words, was derived to reflect the research hotspots in the discipline fields. Bigger nodes represent higher importance of items. A shorter distance indicates stronger relation between nodes. The thicker of the line represents more co-occurrence between two keywords [32].

## Results

### Research areas, publishing trends, and citation index

Among the top 100 most influential articles, 32 were narrative reviews, 16 systematic reviews and/or meta-analysis, 44 clinical research, and 8 basic research. The modifiable factors that drew the most attention were diet or nutrition management in 63 papers, including plant-based diets, dietary sodium restriction, Mediterranean diet, dietary cadmium intake, red meat, high dietary acid load, high protein diet, among others, followed by obesity or body mass index (BMI; *n* = 27), and physical activity or exercises (*n* = 8). Other factors being investigated that obtain high citations included smoking, alcohol consumption, and lipid. The outcomes of interest were the risk of the incidence and progression of CKD, including, in three articles, kidney transplantation, as well as the management and prevention of adverse outcomes associated with CKD. Based on original information on research areas retrieved from the SCIE database, 65 papers were classified into urology and nephrology, 16 in nutrition dietetics, 10 in general internal medicine, 10 in transplantation, and 7 in endocrinology metabolism. The characteristics and methodology of the included top 20 and top 100-cited articles, and summarized authors’ views on the impact of modifiable lifestyles on CKD were listed in Table 1 and Table S3 respectively, ordered by descending adjusted citation index.

**Table 1:** Bibliometric information associated with the top 20 of the top 100-cited articles in lifestyle and CKD

Rank^a^	Article title	Journal	Research method	Publication year	Adjusted citation index^b^	Total cited times, WoS Core	DOI
1	Obesity-Induced Hypertension Interaction of Neurohumoral and Renal Mechanisms	Circulation Research	Narrative review	2015	72.7	436	10.1161/CIRCRESAHA.116.305697
2	Obesity, Oxidative Stress, Adipose Tissue Dysfunction, and the Associated Health Risks: Causes and Therapeutic Strategies	Metabolic Syndrome and Related Disorders	Narrative review	2015	48.5	291	10.1089/met.2015.0095
3	Nutritional Management of Chronic Kidney Disease	New England Journal of Medicine	Narrative review	2017	47.8	191	10.1056/NEJMra1700312
4	Obesity, kidney dysfunction and hypertension: mechanistic links	Nature Reviews Nephrology	Narrative review	2019	45.0	90	10.1038/s41581-019-0145-4
5	Obesity in the critically ill: a narrative review	Intensive Care Medicine	Narrative review	2019	35.5	71	10.1007/s00134-019-05594-1
6	Metabolically Healthy Obesity and Development of Chronic Kidney Disease A Cohort Study	Annals of Internal Medicine	Clinical research	2016	30.4	152	10.7326/M15-1323
7	Fatty kidney: emerging role of ectopic lipid in obesity-related renal disease	Lancet Diabetes & Endocrinology	Narrative review	2014	26.1	183	10.1016/S2213-8587(14)70065-8
8	A Comparison of Treating Metabolic Acidosis in CKD Stage 4 Hypertensive Kidney Disease with Fruits and Vegetables or Sodium Bicarbonate	Clinical Journal of the American Society of Nephrology	Clinical research	2013	25.4	203	10.2215/CJN.02430312
9	Plant-Dominant Low-Protein Diet for Conservative Management of Chronic Kidney Disease	Nutrients	Narrative review	2020	25.0	25	10.3390/nu12071931
10	Treatment of metabolic acidosis in patients with stage 3 chronic kidney disease with fruits and vegetables or oral bicarbonate reduces urine angiotensinogen and preserves glomerular filtration rate	Kidney International	Clinical research	2014	24.6	172	10.1038/ki.2014.83
11	Plant-Based Diets and Incident CKD and Kidney Function	Clinical Journal of the American Society of Nephrology	Clinical research	2019	24.0	48	10.2215/CJN.12391018
12	Dietary Cadmium Intake and Its Effects on Kidneys	Toxics	Narrative review	2018	23.7	71	10.3390/toxics6010015
13	Ketoanalogue-Supplemented Vegetarian Very Low-Protein Diet and CKD Progression	Journal of the American Society of Nephrology	Clinical research	2016	23.2	116	10.1681/ASN.2015040369
14	Lipid Accumulation and Chronic Kidney Disease	Nutrients	Narrative review	2019	23.0	46	10.3390/nu11040722
15	The Role for Protein Restriction in Addition to Renin-Angiotensin-Aldosterone System Inhibitors in the Management of CKD	American Journal of Kidney Diseases	Narrative review	2019	23.0	46	10.1053/j.ajkd.2018.06.016
16	Sodium Intake, ACE Inhibition, and Progression to ESRD	Journal of the American Society of Nephrology	Clinical research	2012	22.9	206	10.1681/ASN.2011040430
17	The Association Between Dietary Sodium Intake, ESRD, and All-Cause Mortality in Patients With Type 1 Diabetes	Diabetes Care	Clinical research	2011	22.4	224	10.2337/dc10-1722
18	Diabetic nephropathy: recent advances in pathophysiology and challenges in dietary management	Diabetology & Metabolic Syndrome	Narrative review	2019	22.0	44	10.1186/s13098-019-0403-4
19	Plant-based diets to manage the risks and complications of chronic kidney disease	Nature Reviews Nephrology	Narrative review	2020	22.0	22	10.1038/s41581-020-0297-2
20	Healthy Dietary Patterns and Risk of Mortality and ESRD in CKD: A Meta-Analysis of Cohort Studies	Clinical Journal of the American Society of Nephrology	Meta-analysis	2017	21.8	87	10.2215/CJN.06190616

^a^ Ranked by adjusted citation index

^b^Adjusted citation index = Total cited times /(2021-Publication year)

**Fig. 2:**
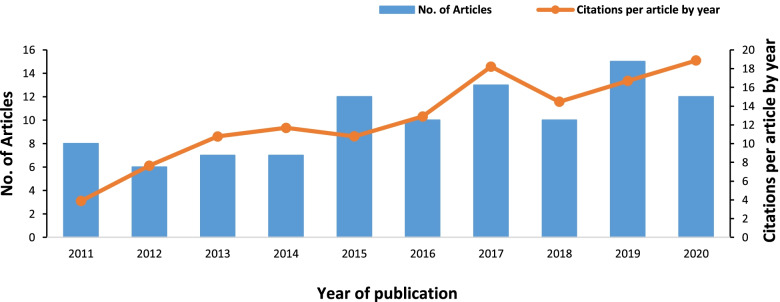
Annual publication numbers and citations per article by year

Despite minor recessions in certain years, the trend analysis demonstrated an annual growth rate of 4.6% in the number of publications across the decade and climbed to the peak in 2019 (*n* = 15; Fig. 2). The H-index was 60 of the retrieved articles. The original citation number of these articles ranged from 12 to 436, with an average citation number of 87.2 per paper. Among the included 48 narrative and systematic reviews/meta-analysis, 28 cited other top highly-cited articles listed in this study, but no duplicated publications were identified. The top three articles receiving the highest citation included one study investigating the interaction between obesity-induced hypertension with neurohumoral and renal mechanisms [33], a review on the association between obesity, oxidative stress, adipose tissue dysfunction, and health risks, including CKD [34], both published in 2015, and a study on the association between dietary sodium intake, ESRD, and mortality in diabetic patients published in 2011 [35]. If adjusted citation index was considered, in the latest three years, the most influential papers were three reviews addressing the impact of obesity and diet nutrition on CKD [36–38].

### Journals, authors, and their countries and institutions

**Fig. 3:**
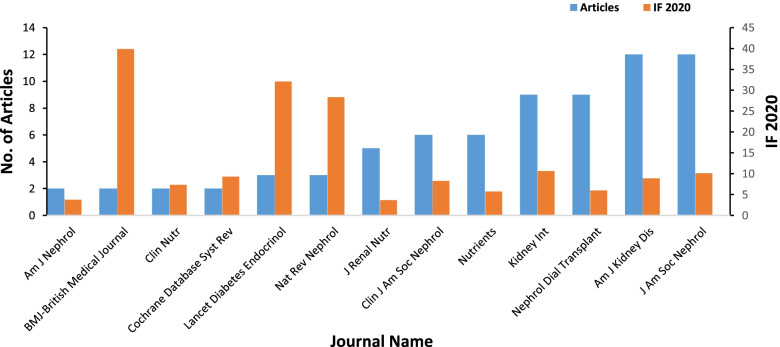
Journals that contribute 2 articles or more to the top 100-cited papers and journal impact factor in 2020. IF, impact factor; Am J Nephrol, American Journal of Nephrology; Clin Nutr, Clinical Nutrition; Cochrane Database Syst Rev., Cochrane Database of Systematic Reviews; Lancet Diabetes Endocrinol, Lancet Diabetes & Endocrinology; Nat Rev. Nephrol, Nature Reviews Nephrology; J Renal Nutr, Journal of Renal Nutrition; Clin J Am Soc Nephrol, Clinical Journal of the American Society of Nephrology; Kidney Int, Kidney International; Nephrol Dial Transplant, Nephrology Dialysis Transplantation; Am J Kidney Dis, American Journal of Kidney Diseases; J Am Soc Nephrol, Journal of the American Society of Nephrology

**Fig. 4:**
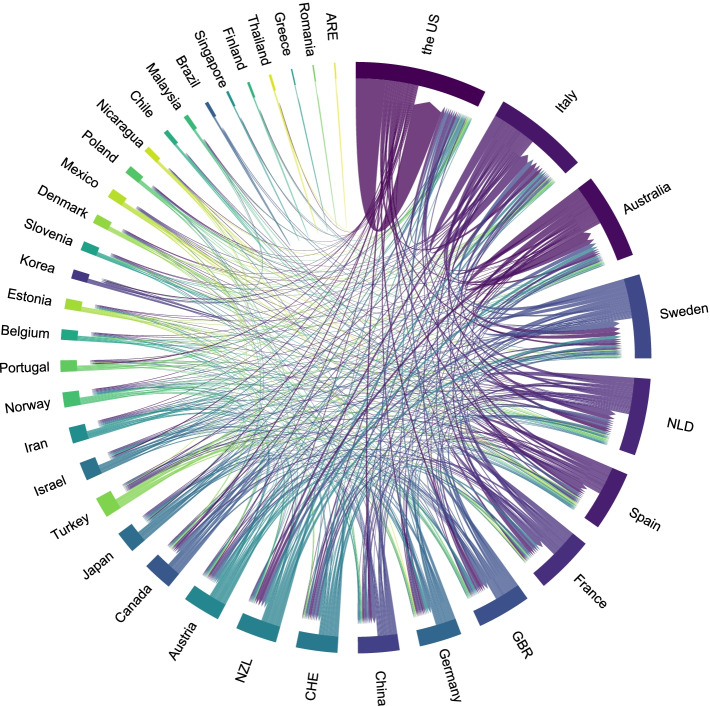
The cooperation relationships of countries that published the top 100-cited articles. The US, the United States; NLD, Netherlands; GBR, the United Kingdom; CHE, Switzerland; NZL, New Zealand; ARE, United Arab Emirates

The top 100-cited articles were published in 40 journals, with 2020 impact factor (IF) ranging between 3.655 to 39.890. The four journals with the most publications were Journal of the American Society of Nephrology (*n* = 12; IF = 10.121), American Journal of Kidney Diseases (*n* = 12; IF = 8.860), and Kidney International (*n* = 9; IF = 10.612) based in the US, and Nephrology Dialysis Transplantation (*n* = 9; IF = 5.992) based in Europe (Fig. 3). The primary corresponding authors of the top 100-cited articles were from 37 countries. Table 2 and Table 3 list the corresponding authors’ countries who contributed to more than two articles and institutions that contributed more than five articles. The US occupied a dominant position in the perspective of article numbers and international partnerships (Fig. 4). Johns Hopkins University in the US was identified as the most productive institution for the highly-cited papers on this topic (*n* = 12), followed by University of California Irvine in the US (*n* = 11), and Karolinska Institute in Sweden (*n* = 8).

**Table 2:** Countries that contribute more than 2 articles in the top 100-cited papers

Country^a^	Articles	Total citations	Average article citations
The US	45	4816	107.0
Australia	9	584	64.9
Italy	5	538	107.6
Netherlands	4	406	101.5
Spain	4	176	44.0
France	4	160	40.0
China	4	146	36.5

^a^According to corresponding authors’ countries. The US, the United States

**Table 3:** Institutions that contributed more than 5 articles in the top 100-cited papers

Affiliations	Country	Articles	Total citations	Average article citations
Johns Hopkins University	The US	12	725	60.4
University of California Irvine	The US	11	872	79.3
Karolinska Institute	Sweden	8	400	50.0
University of Sydney	Australia	7	387	55.3
University of Groningen	Netherlands	6	494	82.3
University of Tennessee	The US	6	422	70.3
University of Bari	Italy	6	329	54.8
University of Lyon	France	6	219	36.5

**Fig. 5:**
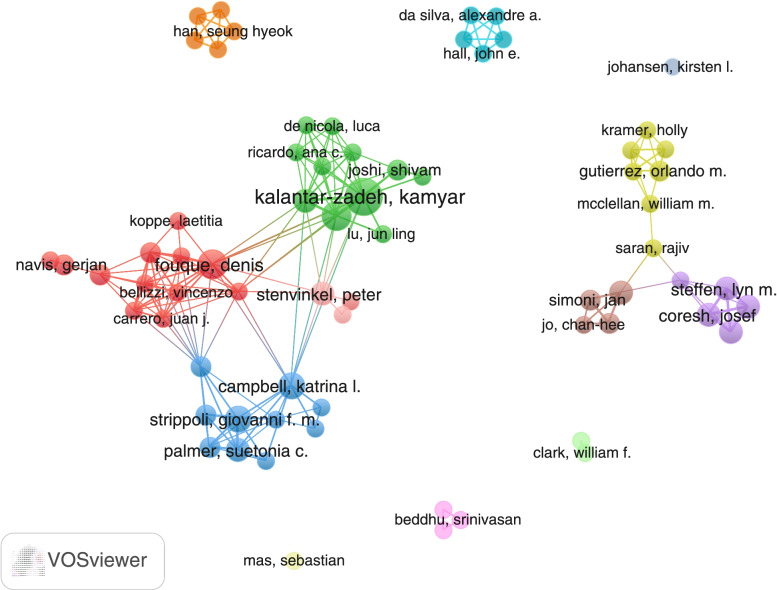
Network visualization map of the co-authorship network for authors in top 100-cited articles

A total of 591 authors contributed to the top 100-cited articles. The top ten researchers contributing to the field are listed in Table 4 based on their number of publications, and Fig. 5 shows the co-authorship network of authors who contributed at least two papers in the top 100-cited articles. The most productive author was, in the US, Kalantar-Zadeh, Kamyar based in University of California Irvine with active collaboration with other scholars, and outside the US, Campbell, Katrina L. based in Princess Alexandra Hospital, Brisbane, Australia. The above-mentioned information of authors’ institutions was based on their publications in 2020.

**Table 4:** Top 10 authors that contribute most articles to the top 100-cited papers

Rank	Author	Articles	Total citations	Average article citations	H-index^a^
1	Kalantar-Zadeh, Kamyar.	11	768	69.8	105
2	Kovesdy, Csaba P.	7	465	66.4	79
3	Campbell, Katrina L.	6	433	72.2	26
4	Strippoli, Giovanni F. M.	6	367	61.2	57
5	Fouque, Denis	6	213	35.5	56
6	Stenvinkel, Peter	5	265	53.0	90
7	Coresh, Josef	5	251	50.2	131
8	Grams, Morgan E.	5	251	50.2	52
9	Rebholz, Casey M.	5	251	50.2	25
10	Palmer, Suetonia C.	5	249	49.8	41

^a^H-index is extracted from Web of Science

### Keywords and research focus

**Fig. 6:**
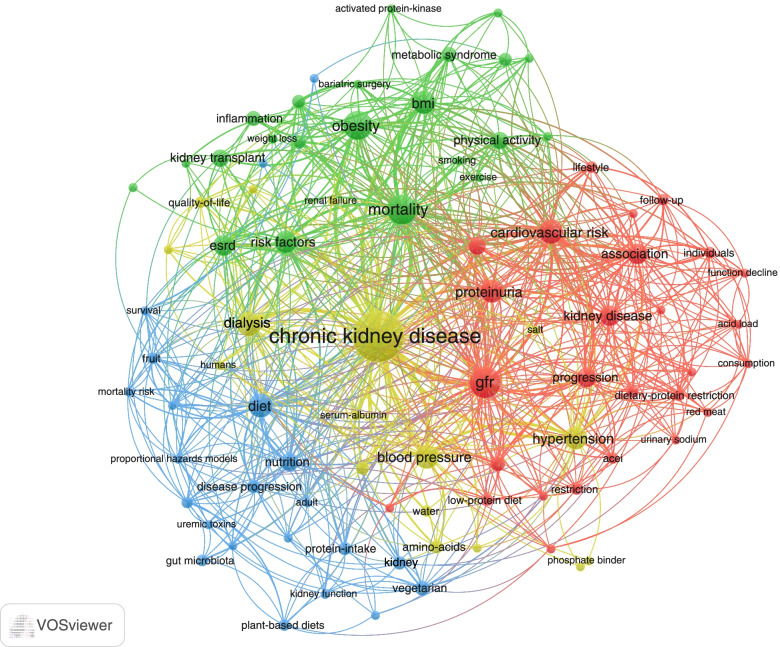
Network visualization map of the keyword co-occurrence network

There were 254 keywords provided by original authors and 494 keywords plus in the top 100-cited articles. Analysis was mainly based on author keywords, except 37 papers in which author keywords were not provided and keywords plus were used instead. The top five keywords with the most frequent occurrence were obesity, diet, blood pressure, BMI, and hypertension; and the top five keywords reflecting outcomes were CKD, glomerular filtration rate, mortality, cardiovascular risk, and dialysis (Fig. S1). Generally, we found diet modification, physical activity, or moderate alcohol consumption was associated with a protective role for the incidence and progression of CKD and its related complications, while obesity or smoking was associated with increased risk for the above-mentioned outcomes (Table S3). Figure 6 presents a co-occurrence network of keywords being listed in at least two papers. They were classified into four clusters, which we assumed to reflect research themes. The leading keywords in the yellow cluster were CKD, dialysis, blood pressure, and hypertension, indicating the focus was mainly on the relationship between blood pressure and CKD, including dialysis. The leading keywords in the red cluster were glomerular filtration rate, cardiovascular risk, proteinuria, kidney disease, association, and progression. The keywords related to lifestyles included dietary protein restriction, low-protein diet, and red meat. We supposed the research focus of the red cluster lied in the impact of protein intake on CKD, especially on kidney function and cardiovascular comorbidities. Leading keywords in the green cluster included mortality, ESRD, kidney transplantation, obesity, BMI, physical activity, and metabolic syndrome. Smoking was also included in the group. Therefore, we assumed the cluster as a group referring to the relation between obesity, physical activities, smoking with the advanced CKD, and adverse outcomes. The leading keyword in the blue cluster was diet, followed by nutrition, vegetarian, protein-intake, disease progression, kidney, gut microbiota, and kidney function. This was considered a concrete cluster that discussed diet and CKD progression.

**Fig. 7:**
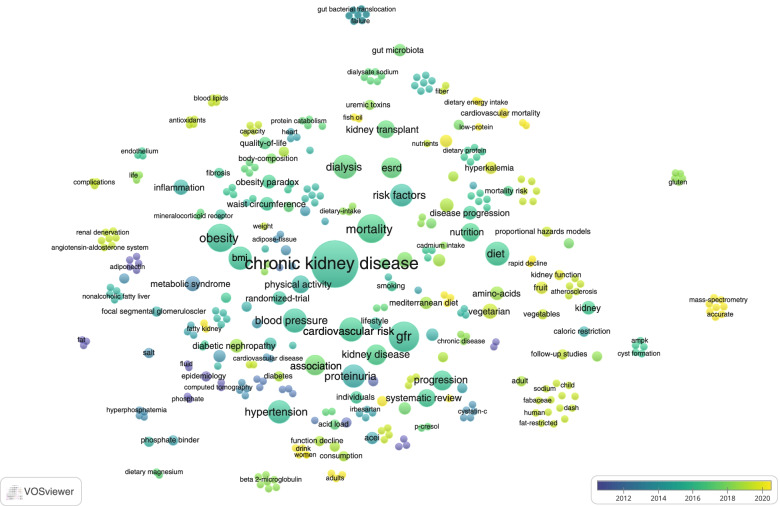
Overlay visualization map of keywords according to the average publication time

In addition, we visualized keywords according to the average publication year to evaluate the trends in the research focus over time. As shown in Fig. 7, the color of the nodes, from purple, blue, green to yellow, corresponds to the earliest to most recent keywords that were used in the publications [39], reflecting which keywords have become popular in recent years and indicating the trend of future hotspots [40]. The nodes for some keywords, ie. dietary sodium, water, and salt were small and colored in purple, indicating these were research topics gaining more popularity a few years ago. Keywords with highly frequent occurrences, such as CKD, physical activity, obesity, diet, nutrition, glomerular filtration rate, progression, mortality, blood pressure, cardiovascular risk, and ESRD were colored in green, we considered these were research topics receiving consistent attention over the decade. We noticed a trend of increasing attention on the gut-kidney axis in the field over 2014 to 2019, with four most-cited papers published [41–44]. Alcohol consumption, fish oil, chain fatty-acids, and water-soluble vitamins, colored in yellow, appeared in 2020 for the first time, indicating the recently emerging research hotspots. Among the newly emerging keywords colored in yellow, words related to diet accounted for a considerable portion.

## Discussion

In this study, we used bibliometric analysis to identify and characterize the top 100-cited articles published between 2011 and 2020 in the field of lifestyle factors’ impact on CKD. Our study provides legible insights on the publishing trends and research themes on the topic. We found about two-thirds of the most cited papers addressing the association between modifiable factors and CKD were clinical research, while basic studies only accounted for a small fraction. Developed countries, especially the US, showed overwhelming influence in this field in terms of the number of top-cited publications. We also noticed the transition of research hotspots over the decade, with diet, nutrition, obesity, and physical activity being the factors constantly drawing attention, and alcohol consumption, gut-kidney axis, fish oil, chain fatty-acids, molecular-weight protein, and water-soluble vitamins being among the newly emerging keywords.

Our finding, that the modifiable factors gaining most popularity were diet or nutritional management, is consistent with the fact that diet contributes substantially to the incidence and progression of CKD, and stays focused in the academic community. Recommendations on protein and sodium intake have been incorporated into guidelines for clinical management of CKD, such as the Kidney Disease: Improving Global Outcomes guideline [1], National Institute for Health and Care Excellence guideline [45], and National Kidney Foundation Kidney Disease Outcomes Quality Initiative guideline [46]. However, as Suetonia C Palmer pointed out, current evidence for dietary interventions in the setting of CKD, with clinical uncertainty, is yet sufficient to guide comprehensive clinical practice [47]. For instance, there are very limited data available evaluating potential adverse effects and participants’ quality of life related to dietary protein restriction [48]. Thus, as indicated in our study, the impact of diet and nutrition on CKD remains an important research topic, and further studies to evaluate the effects of nutritional interventions in the general population for the prevention of incident CKD and in CKD participants for slowing the progression to ESRD are required [48].

Our study showed obesity and health-related behaviors, such as physical activity and smoking, were among the research hotspots of modifiable factors. This evidence supports the inclusion of advice on physical activity, healthy weight, and smoking cessation into CKD management guidelines [1]. This reflects the attention from the field of nephrology on the influence of emerging obesity issues and unhealthy behavioral factors on health outcomes. Both obesity and sedentary lifestyle have become major driving forces for global disease burdens [49–51]. Their associations with CKD are investigated intensely by scholars. For example, in the top-cited articles included in the study, obesity is associated with increased CKD risk, and obese or overweight CKD patients are suggested to maintain a healthy weight and lifestyle [14]. A study evaluated the risk of ESRD associated with obesity at the time of donation among live kidney donors and found that obese live kidney donors have a significant 86% increased risk of ESRD compared to non-obese donors [52]. Regular physical activity instead of sedentariness can reduce the risk and mortality of CKD in type 2 diabetes [53]. A randomized clinical trial found that dietary calorie restriction and aerobic exercise can improve the metabolic milieu in patients with moderate to severe CKD [54]. Besides, a low-intensity exercise program may improve physical performance and quality of life in dialysis patients [55]. Studies suggest that cigarette smoking is an independent risk factor for incident CKD [56, 57], and nonsmoking is associated with a lower risk of adverse outcomes in CKD patients [58] and all-cause mortality [59].

It is interesting to investigate the evolution of research hotspots over time. For example, water intake and dietary sodium were factors receiving high citation years ago. A cross-sectional analysis of the National Health and Nutrition Examination Survey found that higher total water intake, particularly plain water, has a protective effect on CKD [60]. Julie Lin had analyzed longitudinal cohort data to fill the research vacancy of the influence of sodium intake on microalbuminuria and estimated glomerular filtration rate decline and found that less sodium intake can reduce the risk for estimated glomerular filtration rate decline [61]. Besides, dietary salt restriction is essential in patients with CKD and hypertension [62]. Nowadays, alcohol consumption, gut-kidney axis, fish oil, chain fatty-acids, and water-soluble vitamins have drawn more attention. Consuming a low or moderate amount of alcohol may lower the risk of developing CKD [63]. Gut microbiota dysbiosis induces gut-derived uremic toxins formation and is associated with CKD progression [64]. A recent study finds that omega-3 polyunsaturated fatty acids supplementation, such as fish oil can reduce cardiovascular mortality in patients on hemodialysis [65]. Short-chain fatty acids, being derived from fiber-rich diets [42], can delay CKD progression [66]. Vitamin K deficiency in patients on dialysis is associated with vascular calcification, bleeding risk, and cardiovascular disease [67]. Diet modification has been receiving persistent attention from scholars as most newly emerging keywords were related to diet. More research is needed to determine the optimal dietary patterns to prevent kidney disease and its progression [68]. Meanwhile, we noticed, certain research hotspot in other academic fields has not drawn as much attention in nephrology yet. For example, sleep, one of the important modifiable lifestyle factors, which was reported to be associated with a wide range of diseases [69], including CKD [18, 70, 71], was not found in the top 100-cited list. The low citation might be caused by the most recent publication time not allowing the papers to be fully cited, or might indicate not so many scholars were dedicated to the research of sleep and its relation to CKD. Lifestyle modification of sleep in CKD patients requires more attention.

The results showed the US was the most productive country on the current topic and with the most active international partnership. Journal of the American Society of Nephrology, American Journal of Kidney Diseases, Kidney International based in the US, and Nephrology Dialysis Transplantation based in Europe were the four journals with the most publications, indicating the US and European were pilots in the research field about the impact of modifiable factors on CKD; while developing countries were not active in producing highly influential research. The disparity of the quantity of academic publications between developing and developed world has long been recognized, which might be attributed to multifaced causes, to name a few, lacking of research capacity in developing countries [72], funding and principal investigator status owned by developed world [73], language and writing barriers, and editorial bias [74]. Considering the disease burden of CKD in developing countries are rising and might be more pronounced than that in developed countries, high-quality research about the impact of modifiable factors on CKD conducted in population from less developed regions, and more cooperations between developed countries and developing countries are required, such that the evidence can be disseminated to these population more precisely.

Our study has many strengths. To our knowledge, this is the first bibliometric analysis of the relationship between modifiable lifestyles and CKD. Our study finds the evolution of hot topics over the decade and provides clues for scholars to choose research themes. However, there are some limitations of our study. First, only English literature was included in the study, so we may fail to capture some landmark articles published in other languages. Second, all data were extracted from the SCIE of Web of Science, thus, we may fail to capture certain related publications provided in other sources. Third, despite we analyzed the top-cited articles in this field representing the research hotspots, we admit certain research topics with few publications due to publication bias [75], may be missed. In addition, ‘obliteration by incorporation’, which represents that the older publications are no longer cited because their findings are common-use and incorporated into the current discipline, is a notable concern in the bibliometric analysis [76]. Thus, we included publications within the last ten years and ranked articles based on an adjusted citation index rather than the number of citations received in the current year.

## Conclusions

In summary, in the bibliometric analysis of the top 100-cited articles addressing the influence of modifiable factors on CKD, our study provides a comprehensive description of publishing trends and research focus over a decade. The association between modifiable factors and CKD has been among the research focus over the decade. While the study hotspots are evolving over time, diet, obesity, and physical activity were factors receiving the most attention in this topic.

## Supplementary Information

**Additional file 1: Supplementary Table 1.** The detailed search strategy.

**Additional file 2: Supplementary Table 2.** Keywords merging details. **Supplementary Table 3.** Bibliometric information of the top 100-cited articles on the impact of modifiable lifestyles on CKD.

**Additional file 3: Figure S1.** Word cloud of keywords of the top 100-cited articles.

## Data Availability

All data generated or analysed during this study are included in this published article [and its supplementary information files].

## Abbreviations

CKD: Chronic kidney disease
ESRD: End-stage renal disease
The US: The United States
SCIE: Science Citation Index Expanded database
IF: Impact factor
