# Supplementary material for: Relationship between modifiable lifestyle factors and chronic kidney disease: a bibliometric analysis of top-cited publications from 2011 to 2020
Source: BMC Nephrol. 2022 Mar 25;23:120. doi: 10.1186/s12882-022-02745-3 (PMC8957172; doi:10.1186/s12882-022-02745-3)
Supplement: Supplementary file 1 — Additional file 1: Supplementary Table 1. The detailed search strategy. [file 12882_2022_2745_MOESM1_ESM.docx]

Table S1. The detailed search strategy

| Database | Terms |
| --- | --- |
| Science Citation Index Expanded | Chronic kidney disease  **#1**  TS=(“Chronic Kidney Failure” OR “Chronic Renal Insufficiency” OR “chronic kidney disease*” OR “renal failure*” OR “kidney failure” OR “renal impairment” OR “kidney impairment” OR “kidney dysfunction” OR “renal dysfunction” OR “reduced renal function” OR “CKD” OR “progressive kidney” OR “Glomerular Filtration Rate” OR “GFR” OR “eGFR” OR Proteinuri* OR Albuminuria OR microalbuminuria OR “End Stage renal disease” OR “ESRD” OR “End-stage Kidney Disease” OR “ESKD” OR dialysis OR “Renal Replacement Therapy”)  Relation  **#2**  TS= (incidence OR development OR progression OR association)  Lifestyle  **#3**  TS= (habit* OR lifestyle* OR Life-style*)  Diet  **#4**  TS= (Diet* OR Food* OR nutrition* OR vegetable* OR meat* OR dairy OR beverage* OR fruit* OR fiber* OR fibre* OR fish* OR salt* OR sodium OR water OR grain$)  Physical activity  **#5**  TS= ((Exercise* OR “physical activit*” OR Walking OR Sedentary OR Sitting OR Screen-Time OR “Computer Games” OR “Video Games” OR “Television”) OR (running OR jogging OR swimming OR sports OR cycling OR aerobic OR yoga OR Tai-Chi OR Qigong)) |

Table S1. Continued.

| Database | Terms |
| --- | --- |
| Science Citation Index Expanded | Alcohol consumption  **#6**  (TS= (alcohol* or alcohol-drinking) AND TS= (dependen* or disorder* or drink* or misuse* or abuse* or consumption$))  Tobacco smoking  **#7**  TS= (Smok* OR cigar* OR Tobacco* OR Nicotiana* OR tabacum OR nicotine OR cannabis)  Sleep  **#8**  TS= (wake* or nap* or nightmare* or hypersom* or shiftwork* Or “Stay up late” Or bedtime or dyssomnias or Sleep* OR “Disturb* sleep” OR insom* OR “sleep quality” OR “lack of sleep” OR “no sleep”)  Obesity  **#9**  TS= (obesity or obese or “weight gain” or “weight loss” or overweight or overeat* or (weight same change) or (bmi same gain) or (bmi same change) or (bmi same loss) or over-weight)  **#1 AND #2 AND (#3 OR #4 OR #5 OR #6 OR #7 OR #8 OR #9)** |
