# Supplementary figures and images for: Relationship between modifiable lifestyle factors and chronic kidney disease: a bibliometric analysis of top-cited publications from 2011 to 2020
Source: BMC Nephrol. 2022 Mar 25;23:120. doi: 10.1186/s12882-022-02745-3 (PMC8957172; doi:10.1186/s12882-022-02745-3)

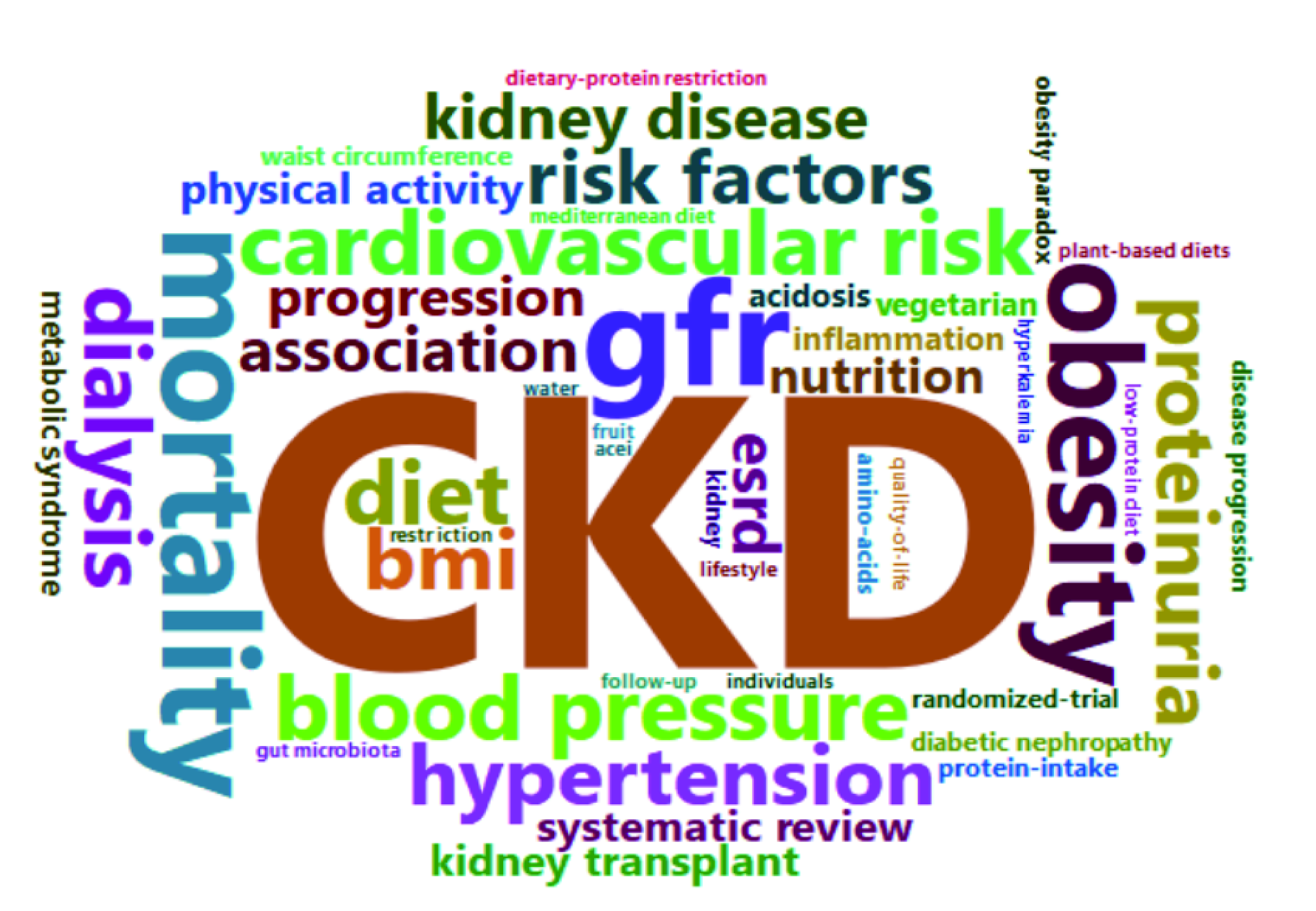

Supplement: Supplementary file 3 — Additional file 3: Figure S1. Word cloud of keywords of the top 100-cited articles. [file 12882_2022_2745_MOESM3_ESM.tif]
